# Percent Body Fat Cut-Off Points for Diagnosing Metabolic Syndrome in Korean Adolescents

**Published:** 2019-01

**Authors:** Saejong PARK, Dong-Sik CHUNG, Byoung-Goo KO, Hong-Sun SONG, Kwang Jun KIM, Jin-Wook CHUNG, Seunghee LEE, Chul-Hyun KIM, Younshin NAM, Seungyun SHIN, Hyo LEE, Sochung CHUNG, Hong-Yup AHN, Jeong Hun OH, Wi-Young SO

**Affiliations:** 1. Department of Sports Sciences, Korea Institute of Sport Science, Seoul, Korea; 2. Department of Sports Culture, Dongguk University, Seoul, Korea; 3. Department of Physical Education, Korea University, Seoul, Korea; 4. Department of Sports Medicine, Soonchunhyang University, Asan, Korea; 5. Department of Sports for All, Duksung Women's University, Seoul, Korea; 6. Department of Martial Arts, Yongin University, Yongin, Korea; 7. Department of Sport and Health Sciences, Sangmyung University, Seoul, Korea; 8. Department of Pediatrics, Konkuk University Medical Center, Research Institute of Medical Science, Konkuk University, School of Medicine, Seoul, Korea; 9. Department of Statistics, Dongguk University, Seoul, Korea; 10. Seoul Physical Education Middle School, Seoul, Korea; 11. Sports and Health Care Major, College of Humanities and Arts, Korea National University of Transportation, Chungju-si, Korea

**Keywords:** Adolescents, Childhood obesity, Percent body fat standards

## Abstract

**Background:**

This study aimed to identify percent body fat cut-off points related to metabolic syndrome in a large sample of Korean adolescents.

**Methods:**

The subjects (n=2120; boys=1107, girls=1013) were middle and high school students aged 12–17 yr who participated in the Korean National Fitness Award Project in 2013. Percent body fat was estimated via eight-polar bioelectrical impedance analysis. Metabolic syndrome was classified using established standards based on the National Cholesterol Education Program with the definition modified for age. Age- and sex-specific percent body fat z-scores were calculated for every adolescent using skewness, median, and coefficient of variation curves to account for growth and development. Receiver operating characteristic curve analysis was used to identify the percent body fat cut-off points using percent body fat z-scores from skewness, median, and coefficient of variation curves as the test and metabolic syndrome as the criterion.

**Results:**

Based on the modified National Cholesterol Education Program criteria for metabolic syndrome, the areas under the receiver operating characteristic curve for percent body fat were 0.882 and 0.893 for boys and girls, respectively. The percent body fat percentiles were 82.2 and 87.3 for boys and girls, respectively. According to the skewness, median, and coefficient of variation curves, the percent body fat cut-off points based on the modified National Cholesterol Education Program criteria were 23.6%–25.7% for boys and 32.8%–37.3% for girls, both aged 12–17 yr. Age- and sex-specific percent body fat cut-off points were identified in relation to the metabolic syndrome status of Korean adolescents.

**Conclusion:**

These percent body fat cut-offs might be useful for identifying metabolic abnormality due to obesity in Korean adolescents.

## Introduction

“Childhood obesity is considered one of the most serious public health problems of the 21st century” (1). It increases the risk of obesity-related health problems in adulthood such as cardiovascular disease and type 2 diabetes (2,3). Because adolescent obesity is strongly related to adult obesity in approximately 80% of cases, the prevention and assessment of childhood obesity are extremely important (4,5).

Obesity is defined as excessive body fat accumulation leading to health problems. Thus, the assessment of body fat is important. Body mass index (BMI), weight (kg) divided by height (m) squared, is used to assess obesity (6); however, relative BMI using age- and sex-specific percentiles from growth charts, rather than absolute BMI, is recommended for the assessment of childhood obesity (7). Although it is feasible to use BMI to assess childhood obesity, in practice, it has a major limitation in its imprecise assessment of adiposity. Specifically, it is limited in its ability to distinguish between fat-free mass and fat mass. Based on the prevalence of cardiovascular disease in the Bogalusa Heart Study, the percent body fat (%BF) standards for defining obesity were identified as 25% BF in boys and 30% BF in girls8). Although %BF cut-off points were identified for defining obesity in American youth (8), it did not account for the variation in %BF during normal growth and maturation (9). Moreover, the reported 85th and 68th %BF-percentile cut-offs for boys and girls, respectively, based on the National Health and Nutrition Examination Survey (NHANES, 1999–2004), can only be applied to American adolescents (10).

Ethnicity may affect the relationships among BMI, %BF, and metabolic syndrome (9,11). However, few international studies have published %BF standards for metabolic risk among youth. Moreover, no such studies for Korean adolescents exist. Therefore, the purpose of this study was to identify %BF cut-off points related to metabolic syndrome in a large sample of Korean adolescents.

## Methods

### Subjects

The subjects (n=2311) were middle and high school students aged 12–17 yr attending Korean National Fitness Centers in Seoul, Daegu, Chungcheongbuk-do, and Jeollanam-do in the Republic of Korea, who participated in the Korean National Fitness Award Project in 2013. All the participants were apparently healthy. Exclusion criteria were health problems such as cardiovascular disease, diabetes, and/or musculoskeletal injuries.

The participants were asked about their willingness to participate in this project. Only willing adolescents participated, and those who were unwilling to participate were excluded from the study.

All study procedures were approved by the Institutional Review Board of the Korea Institute of Sports Science. Written informed consent was received from participants and their parents prior to the study.

### Anthropometry

Body weight was measured using an electronic scale (Inbody 720, Biospace, Seoul, Korea) to the nearest 0.1 kg. Height was measured with a stadiometer to the nearest 0.1 cm. BMI was calculated as weight (kg) divided by height (m) squared.

%BF was evaluated using eight polar bioelectrical impedance frequencies (Inbody 720, Biospace, Seoul, Korea). The validity of the measurements of this device for %BF in youth has previously been published (12). Prior to the measurements, all participants were asked to fast overnight for 8 h, void their bladder prior to the measurement, wear light clothing, and remove all metal items that could interfere with the electronic current during the measurement. All measurements followed published recommendations (13). Furthermore, the reliability of %BF using the In-body 720 device was assessed with a subgroup of the sample who underwent repeated testing, twice within 10 min. The %BF error rates were 2.8% for boys and 1.4% for girls.

### Metabolic Syndrome Risk Factors

Metabolic syndrome was classified using the established standards based on the modified National Cholesterol Education Program (NCEP; Adult Treatment Panel-III) criteria, with the definition modified for age (modified NCEP) (14,15). Participants had metabolic syndrome if the total criteria value was >3: central adiposity (≥90th waist circumference percentile according to age and sex), triglyceride level >110 mg/dL, high-density lipoprotein (HDL) cholesterol level <40 mg/dL, high blood pressure (≥90th systolic or diastolic percentile according to age, sex, and height), and fasting plasma glucose level >100 mg/dL. Blood pressure and waist circumference status were classified using the 2007 Korean National Growth Charts according to age, sex, and height (16).

Waist circumference was measured using a tape-line (Gullick, Japan) positioned on the narrowest part between the ribs and superior iliac crest and measured at the end of a breath in 0.1-cm units. Waist circumference was measured twice, and the average value was recorded.

Blood pressure was measured using an automatic sphygmomanometer (SpaceLabs Medical, model 90207, USA). After a 10-min rest, blood pressure was measured twice with the cuff on the brachial artery.

Further, 10 mL blood was collected from all participants after an 8-hour fast, using a disposable syringe, by a medical technologist under pediatrician supervision. Medication status (antithrombotic or aspirin) and past experience (dizziness) were reviewed to prevent side effects during blood collection (e.g., dizziness). Blood collection was postponed or canceled when it appeared dangerous. If dizziness occurred during the blood collection, the subject was asked to rest until he or she stabilized. The collected blood was centrifuged immediately and kept in a refrigerator at −80 °C. Blood glucose, triglyceride, and HDL cholesterol levels were analyzed using an enzymatic and colorimetric test (AU-680, Beckman Coulter, USA). Glucose was analyzed using the colorimetric (GOD-POD) method with a glucose kit (USA). Triglycerides were analyzed using the enzymatic GPO-POD method and a triglyceride kit (USA). HDL cholesterol was analyzed using an enzymatic and colorimetric test with an HDL cholesterol kit (USA).

### Skewness, Median, and Coefficient of Variation and Receiver Operating Characteristic

Age- and sex-specific %BF z-scores were calculated for every adolescent using LMS (L=skewness, M=median, and S=coefficient of variation) curves to account for growth and development (17). LMS curves were calculated separately for boys and girls using LMS ChartMaker Light version 2.54 (Medical Research Council, London, UK) (17). Receiver operating characteristic (ROC) curve analyses were used to identify the %BF cut-off points using %BF z-scores from the LMS methods for the metabolic syndrome criteria (modified NCEP). The ROC analyses were performed only for gender (i.e., boys and girls), not for individual age groups, as age was factored into the %BF z-scores using the LMS method to increase statistical power. The ROC curve represents the changes in sensitivity (true positive rate) and specificity (true negative rate) in the range of potential cut-off values using the diagnostic test. The area under the ROC curve (AUC) is used as a quantitative measure of the accuracy of the diagnostic test to classify the populations into separate groups: non-informative (AUC=0.5), less accurate (0.6<AUC ≤0.7), moderately accurate (0.7<AUC ≤0.9), highly accurate (0.9<AUC<1.0), and perfect discriminatory test (AUC=1.0) (18). The AUC indicated the ability of %BF z-scores to categorize adolescents into a group with or without metabolic syndrome.

### Statistical Analysis

All data are represented as the mean (standard deviation). All analyses were conducted using SPSS version 18.0 (Chicago, IL, USA), except for the LMS. Age- and sex-specific %BF using the smoothed LMS methods were analyzed using LMS ChartMaker Light version 2.54 (Medical Research Council, London, UK). ROC analyses were performed using MedCalc version 14.12.0 (MedCalc Software, Ostend, Belgium). Statistical significance was accepted for values of *P*<0.05.

## Results

One hundred ninety-one individuals who were unwilling to participate were excluded, and the analyzed sample comprised 2120 participants (boys=1107; girls=1013). The participants’ characteristics and prevalence of metabolic syndrome are listed in Table 1. Mean BMI was at the 50th percentile compared to the Korean National Growth Charts for both sexes 14). The prevalence of metabolic syndrome were 3.2% and 2.2% for boys and girls, respectively, according to the modified NCEP standards.

**Table 1:** Participant characteristics

***Variable***	***Boys (n = 1,107)***	***Girls (n = 1,013)***
Characteristics, M (SD)		
Age (yr)	14.6 (1.6)	14.9 (1.6)
Height (cm)	169.6 (7.7)	159.8 (5.1)
Weight (kg)	61 (13.3)	52.8 (8.3)
Body mass index (kg/m ^ 2 ^ )	21.1 (3.8)	20.7 (3)
Body fat (%)	17.8 (7.5)	29.2 (5.7)
Age (yr)		
12	19.08 (8.14)	26.33 (5.85)
13	17.76 (7.88)	27.21 (5.65)
14	17.92 (7.72)	29.10 (5.58)
15	17.25 (6.90)	29.12 (5.79)
16	17.87 (7.54)	30.17 (4.99)
17	17.13 (6.83)	31.33 (5.28)
Prevalence of risk factors ^A^ , % (n)		
High waist circumference	5.9 (55)	6.5 (64)
High blood pressure	25.8 (282)	26.3 (266)
High triglycerides	19 (187)	17.4 (165)
Low HDL cholesterol	14.7 (144)	12.8 (122)
High fasting glucose	0.2 (2)	0.5 (5)
Metabolic syndrome	3.2 (27)	2.2 (20)

A. National Cholesterol Education Program (Adult Treatment Panel III) definition modified for age HDL, high-density lipoprotein

**Fig. 1:**
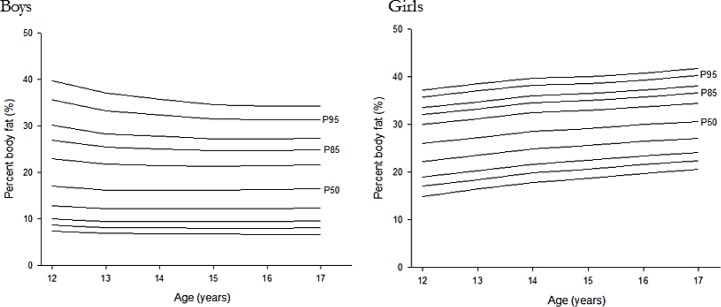
Smoothed LMS curves for Korean adolescents. LMS, L = skewness, M = median, and S = coefficient of variation

The smoothed LMS curves for select %BF percentiles for Korean adolescents are represented in Table 2 and Fig. 1.

**Table 2:** Smoothed LMS curves for selected percentiles of percent body fat for Korean adolescents

***Age (yr)***	***L***	***M***	***S***	***2nd***	***5th***	***10th***	***25th***	***50th***	***75th***	***85th***	***90th***	***95th***	***97th***
Boys (n=1,107)													
12 (n=85)	−0.108	17.087	0.430	7.36	8.65	10.01	12.85	17.09	22.94	26.97	30.14	35.63	39.79
13 (n=230)	−0.069	16.250	0.426	6.95	8.20	9.51	12.23	16.25	21.72	25.44	28.35	33.32	37.04
14 (n=220)	−0.012	16.198	0.420	6.87	8.14	9.47	12.21	16.20	21.51	25.06	27.79	32.41	35.82
15 (n=222)	0.066	16.161	0.415	6.73	8.04	9.41	12.19	16.16	21.32	24.69	27.25	31.49	34.57
16 (n=208)	0.124	16.312	0.413	6.66	8.02	9.44	12.29	16.31	21.45	24.75	27.23	31.31	34.24
17 (n=142)	0.135	16.447	0.411	6.71	8.09	9.52	12.40	16.45	21.59	24.89	27.36	31.41	34.32
Girls (n=1,013)													
12 (n=67)	0.817	26.113	0.219	14.9	17.0	19.0	22.3	26.1	30.0	32.2	33.6	35.8	37.3
13 (n=168)	0.698	27.272	0.208	16.4	18.5	20.3	23.5	27.3	31.2	33.3	34.8	37.1	38.5
14 (n=197)	0.668	28.589	0.197	17.8	19.8	21.7	24.9	28.6	32.5	34.6	36.1	38.3	39.8
15 (n=214)	0.698	29.219	0.187	18.7	20.7	22.5	25.6	29.2	33.0	35.1	36.5	38.6	40.0
16 (n=195)	0.639	30.067	0.179	19.8	21.7	23.5	26.5	30.1	33.8	35.8	37.2	39.4	40.8
17 (n=172)	0.489	30.691	0.177	20.6	22.4	24.1	27.1	30.7	34.5	36.6	38.1	40.3	41.8

LMS, L = skewness, M = median, and S = coefficient of variation

**Fig. 2:**
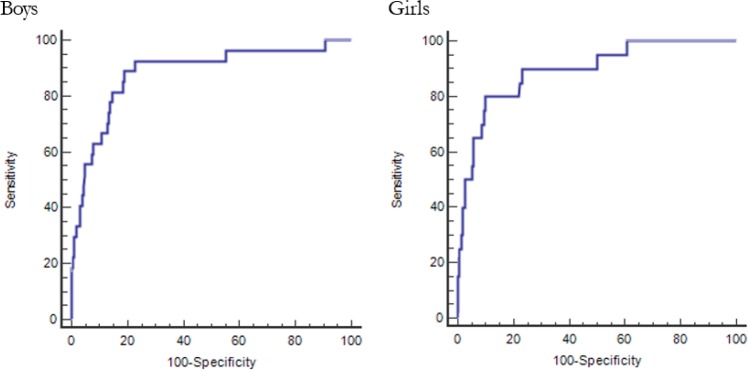
Receiver operating characteristic curves for percent body fat cut-off points for diagnosing metabolic syndrome in Korean adolescents

From the ROC analyses, the AUC values of the %BF z-scores for metabolic syndrome based on the modified NCEP were 0.882 and 0.893 for boys and girls, respectively (Table 3 and Fig. 2). These AUC values implied that %BF z-scores are moderately accurate at identifying metabolic syndrome based on the modified NCEP criteria.

**Table 3:** Percent body fat cut-off points associated with metabolic syndrome for Korean adolescents

***Variable***	***Percent body fat cut-off points***	
	***Boys***	***Girls***
Age (yr)		
12	25.7	32.8
13	24.3	34.0
14	23.9	35.3
15	23.6	35.7
16	23.7	36.5
17	23.9	37.3
Percent body fat percentile	82.2th	87.3th
Sensitivity (95% CI)	88.89 (70.8 – 97.6)	80.00 (56.3 – 94.3)
Specificity (95% CI)	80.98 (78.1 – 83.6)	89.82 (87.6 – 91.7)
Area under the curves	0.882	0.893

CI, confidence interval; ROC, receiver operating characteristic

The ROC analysis based on metabolic syndrome using the modified NCEP resulted in %BF percentile cut-offs of 82.2 and 87.3 for boys and girls, respectively. According to the LMS curves, the %BF cut-off points based on the modified NCEP ranged from 23.6% to 25.7% for boys and 32.8% to 37.3% for girls (Table 3).

## Discussion

To the best of our knowledge, this is the first study to identify age- and sex-specific %BF cut-off points for Korean adolescents related to health outcome standards as well as metabolic syndrome. The %BF z-score and pediatric definition of metabolic syndrome were used to account for changes in adolescents due to growth. Although longitudinal data would be ideal for describing such changes, these cross-sectional data will help to facilitate early identification of adolescents at risk and serve as basic data for future research.

Western countries use a BMI >30 kg/m^2^ to define obesity, whereas Asian countries use BMI >25 kg/m^2^, because body fat composition differs between Asian and Western countries, although sex and BMI also affect the result, in addition to race, region, lifestyle, diet, and genetics (19,20). Using ROC analyses, a well-designed study of American youth reported that %BF is moderately accurate (AUC=0.885 in boys and 0.836 in girls) at differentiating adolescents with and without metabolic syndrome (10). The present %BF thresholds were used to identify adolescents with metabolic syndrome and had similar AUC values (0.882 and 0.893 for boys and girls, respectively, using the modified NCEP criteria).

%BF cut-off points for metabolic syndrome were 31.5%–35.9% and 35.5%–38.6% for boys and girls aged 12–18.9 yr, respectively (10). Another study reported %BF thresholds of 28.7% for men and 37.2% in women, using the diagnostic definition of metabolic syndrome for adults (21). Although the %BF cut-off points for Korean girls were similar to those reported by previous studies, the %BF cut-off points for Korean boys were considerably different. Our results suggested that it may be ideal to use cut-off values for metabolic syndrome of 23.6%–25.7% and 32.8%–37.3% for male and female adolescents aged 12–17, respectively, based on the modified NCEP criteria.

The limitations of this study are as follows. First, this was a cross-sectional study. %BF thresholds should be determined in a prospective cohort study that predicts adult health outcomes from %BF in youth. This study used percentiles and z-score references as alternatives to account for potential differences by maturation, but these references cannot substitute for longitudinal analyses that assess maturity. Second, the use of a bioelectrical impedance frequency method for %BF estimation might have resulted in error. Nevertheless, the strength of this study is that it suggests %BF cut-off points for Korean adolescents for the prediction of metabolic syndrome according to gender and age. This study provides precise information about %BF cut-off points in Asia used as criteria for Asian participants in other studies across various countries. In addition, this study used a large national sample of adolescents, reference percentiles to summarize the differences in %BF and definition of metabolic syndrome according to age and gender, and ROC curves that evaluate %BF cutoff points from the modified NCEP criteria. Further, this study provides evidence that maintaining a healthy %BF is helpful for reducing the risk of metabolic syndrome in Korean adolescents. This information can be used as basic data by policymakers for adolescent obesity management and prevention, as well as by those identifying future national criteria for metabolic syndrome.

## Conclusion

Age- and sex-specific %BF cut-off points were identified in relation to metabolic syndrome status in Korean adolescents. The sex-specific %BF percentiles were similar in their ability to identify metabolic syndrome as the modified NCEP criteria. These %BF cut-off values might be a useful tool for identifying metabolic abnormality due to obesity among Korean adolescents.

## Ethical considerations

Ethical issues (Including plagiarism, informed consent, misconduct, data fabrication and/or falsification, double publication and/or submission, redundancy, etc.) have been completely observed by the authors.

## References

[1] World Health Organization (2016). Childhood overweight and obesity. World Health Organization.

[2] Freedman D Khan LK Serdula MK (2005). The relation of childhood BMI to adult adiposity: The Bogalusa Heart Study. Pediatrics, 115( 1): 22– 27. 15629977 10.1542/peds.2004-0220

[3] Whitaker RC Wright JA Pepe MS (1997). Predicting obesity in young adulthood from childhood and parental obesity. N Engl J Med, 337( 13): 869– 873. 9302300 10.1056/NEJM199709253371301

[4] Kvaavik E Tell GS Klepp KI (2003). Predictors and tracking of body mass index from adolescence into adulthood: follow-up of 18 to 20 years in the Oslo Youth Study. Arch Pediatr Adolesc Med, 157( 12): 1212– 1218. 14662578 10.1001/archpedi.157.12.1212

[5] Daniels SR Arnett DK Eckel RH (2005). Overweight in children and adolescents path-ophysiology, consequences, prevention, and treatment. Circulation, 111( 15): 1999– 2012. 15837955 10.1161/01.CIR.0000161369.71722.10

[6] National Institute of Health (1998). Clinical guidelines on the identification, evaluation, and treatment of overweight and obesity in adults. Obes Res, 6 Suppl 2: 51S– 209S. 9813653

[7] Barlow SE (2007). Expert committee recommendations regarding the prevention, assessment, and treatment of child and adolescent overweight and obesity: summary report. Pediatrics, 120 Suppl 4: S164– S192. 18055651 10.1542/peds.2007-2329C

[8] Williams DP Going SB Lohman TG (1992). Body fatness and risk for elevated blood pressure, total cholesterol, and serum lipoprotein ratios in children and adolescents. Am J Public Health, 82( 3): 358– 363. 1536350 10.2105/ajph.82.3.358 PMC1694353

[9] Laurson KR Eisenmann JC Welk GJ (2011). Body fat percentile curves for US children and adolescents. Am J Prev Med, 41( 4 Suppl 2): S87– 92. 21961617 10.1016/j.amepre.2011.06.044

[10] Laurson KR Eisenmann JC Welk GJ (2011). Aerobic fitness percentiles for US adolescents. Am J Prev Med, 41( 4 Suppl 2): S106– 110. 21961609 10.1016/j.amepre.2011.07.005

[11] Deurenberg P Deurenberg-Yap M Guricci S (2002). Asians are different from Caucasians and from each other in their body mass index/body fat per cent relationship. Obes Rev, 3( 3): 141– 146. 12164465 10.1046/j.1467-789x.2002.00065.x

[12] Lim JS Hwang JS Lee JA (2009). Cross-calibration of multi-frequency bioelectrical impedance analysis with eight-point tactile electrodes and dual-energy X-ray absorptiometry for assessment of body composition in healthy children aged 6–18 years. Pediatr Int, 51( 2): 263– 268. 19405930 10.1111/j.1442-200X.2008.02698.x

[13] Heyward VH Wagner DR (2004). Applied body composition assessment; Human Kinetics.

[14] Cook S Weitzman M Auinger P (2003). Prevalence of a metabolic syndrome pheno-type in adolescents: findings from the third National Health and Nutrition Examination Survey, 1988–1994. Arch Pediatr Adolesc Med, 157( 8): 821– 827. 12912790 10.1001/archpedi.157.8.821

[15] Ford ES Li C (2008). Defining the metabolic syndrome in children and adolescents: will the real definition please stand up? J Pediatr, 152( 2): 160– 164. 18206681 10.1016/j.jpeds.2007.07.056

[16] Moon JS Lee SY Nam CM (2008). 2007 Korean National Growth Charts: review of developmental process and an outlook (in Korean). Korean J Pediatr, 51( 1): 1– 25.

[17] Park S Song H-S Ko B-G (2013). Body composition and metabolic syndrome in Korean Adolescents (in Korean). Korea Institute of Sport Science.

[18] Obuchowski NA (2003). Receiver Operating Characteristic Curves and Their Use in Radiology. Radiology, 229( 1): 3– 8. 14519861 10.1148/radiol.2291010898

[19] World Health Organization (2000). International association for the study of obesity and International obesity task force: The Asia-Pacific Perspective: redefining obesity and its treatment. Health Communications Australia: Melbourne.

[20] Wadden TA Stunkard AJ (2002). Handbook of obesity treatment. Guilford Press.

[21] Zhu S Wang Z Shen W (2003). Percentage body fat ranges associated with metabolic syndrome risk: results based on the third National Health and Nutrition Examination Survey (1988–1994). Am J Clin Nutr, 78( 2): 228– 235. 12885702 10.1093/ajcn/78.2.228

